# AGENCY AS IDEOLOGY: PRECARITY AND DISEMBEDDEDNESS IN THE 21ST CENTURY LIFE COURSE

**DOI:** 10.1093/geroni/igz038.2184

**Published:** 2019-11-08

**Authors:** wenxuan huang

**Affiliations:** Department of Sociology, Case Western Reserve University, Cleveland, Ohio, United States

## Abstract

The “individualization” thesis has gradually merged into the discussion of increasing heterogeneity of the life course as well as growing inequality over historical time. As individuals are “disembedded” from both cultural traditions and more recently social institutions, individual agency has drawn revived interest in outlining “choice biography” that is seen as paramount to personal outcomes and even containing overcoming force against structure. This practice mutes the consideration of the ongoing forces of social structure that by their very nature continue to constitute individual selves and possibilities. The uncritical treatment of individual agency makes it problematic for the study of precarity, mystifying and obscuring the analysis of inequality-generating mechanisms, reducing them to the individual-level. We analyze current uses of the concept of agency in the life course research, and particularly in the areas of transition research, e.g., transition to adulthood/retirement, where individual agency is assumed to be most active.

